# Utilization and Predictors of Electrical Cardioversion in Patients Hospitalized for Atrial Fibrillation

**DOI:** 10.1155/2016/8956020

**Published:** 2016-02-07

**Authors:** Yogita M. Rochlani, Nishi N. Shah, Naga V. Pothineni, Hakan Paydak

**Affiliations:** ^1^Department of Internal Medicine, University of Arkansas for Medical Sciences, Little Rock, AR 72205, USA; ^2^Division of Cardiology, University of Arkansas for Medical Sciences, Little Rock, AR 72205, USA

## Abstract

Atrial fibrillation (AF) is a common arrhythmia in adults associated with thromboembolic complications. External electrical cardioversion (DCCV) is a safe procedure used to convert AF to normal sinus rhythm. We sought to study factors that affect utilization of DCCV in hospitalized patients with AF. The study sample was drawn from the Nationwide Inpatient Sample (NIS) of the Healthcare Cost and Utilization Project in the United States. Patients with a primary discharge diagnosis of AF that received DCCV during hospitalization in the years 2000–2010 were included. An estimated 2,810,530 patients with a primary diagnosis of AF were hospitalized between 2001 and 2010, of which 1,19,840 (4.26%) received DCCV. The likelihood of receiving DCCV was higher in patients who were males, whites, privately insured, and aged < 40 years and those with fewer comorbid conditions. Higher CHADS2 score was found to have an inverse association with DCCV use. In-hospital stroke, in-hospital mortality, length of stay, and cost for hospitalization were significantly lower for patients undergoing DCCV during AF related hospitalization. Further research is required to study the contribution of other disease and patient related factors affecting the use of this procedure as well as postprocedure outcomes.

## 1. Introduction

Atrial fibrillation (AF) is the most common clinically encountered arrhythmia in adults. The estimated prevalence of AF is expected to increase from 5.2 million in 2010 to 12.1 million by 2030 [1]. AF related hospitalization has increased by 23% between 2000 and 2010, and the cost of care associated with this diagnosis is also noted to be on the rise [2]. Electrical cardioversion (DCCV) with concomitant antiarrhythmic therapy is a safe and effective technique to achieve conversion to normal sinus rhythm in patients with AF [3]. Age, gender, and race related differences in the prevalence and outcomes of AF have been studied. There is limited literature on the impact of these demographic factors on the treatments and procedures offered to patients with AF. Data regarding utilization of DCCV as a procedural treatment for AF in the United States is scarce. In this study, we describe trends in utilization of DCCV as a procedural treatment for AF in the United States over a 10-year period, explore demographics related disparities in utilization of DCCV for AF, identify predictors for DCCV use in a real world setting, and assess the impact of DCCV use on outcomes.

## 2. Methods

The Nationwide Inpatient Sample (NIS) contains data on hospital inpatient stays from states participating in the Healthcare Cost and Utilization Project (HCUP). Data for a single year includes information on approximately 8 million inpatient stays from about 1,000 hospitals. We used data from the years 2001–2010, a ten-year-long period. We identified adult patients hospitalized for AF using the primary discharge diagnosis and further identified those that underwent DCCV during the hospital stay using ICD-9 codes. For the demographic characteristics, proc surveyfreq and proc surveymeans were used taking the sample design into account. For logistic regression, we used proc surveylogistic. Demographic variables used for our analyses include gender (female (reference group)), race (whites (reference group), blacks, and others), and age (<40 years (reference group), 40–64 years, 65–74 years, and 75 years and above). Comorbidities such as diabetes, congestive heart failure, and hypertension were identified using appropriate ICD-9 codes and accounted for. Outcomes with regard to length of stay [LOS], in-hospital mortality, cost of hospitalization, and postoperative stroke were measured. Descriptive statistics and outcome measurements were calculated using univariate analysis. Multivariate logistic regression was used for Table 2 to describe predictors for utilization of DCCV and backward elimination method was used to derive the final model for multivariate logistic regression. All statistical analyses were conducted using SAS version 9.3 (SAS, Cary, North Carolina). A *p* value of less than 0.05 was considered statistically significant.

## 3. Results

**Figure 1:**
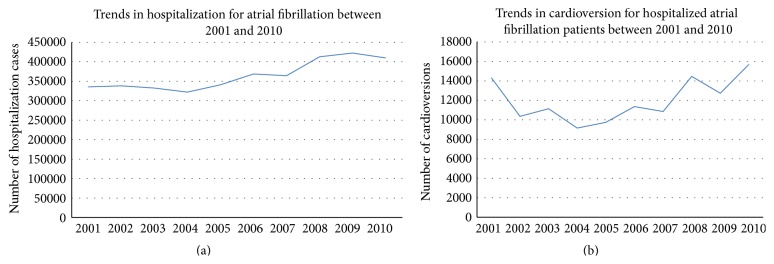


There were a total of 2,810,530 patients hospitalized with a primary diagnosis of AF between 2001 and 2010, of which 119,840 (4.26%) patients underwent inpatient DCCV. Though AF related hospitalization has been increasing, trends in utilization of DCCV have remained stable (Figure 1). There were significant age related disparities in the utilization of DCCV with the procedure being performed the most in patients between 41 and 64 years of age (Table 1) and least commonly in patients over 75 years of age. DCCV rates were higher in men as compared with women (58.4% versus 41.6%). Among patients who received DCCCV, 86.4% were Caucasian, 5.9% were African American, and 7.7% belonged to other races. 37% of patients receiving DCCV were privately insured. 53.2% had Medicare, 5.1% had Medicaid, and 4.6% were uninsured. Patients who underwent DCCV had significantly lower prevalence of diabetes (17.8% versus 20.9%, *p* < 0.001), hypertension (59.1% versus 61.7%, *p* < 0.001), and congestive heart failure (13.4% versus 14.7%, *p* < 0.001) when compared with those who did not. A third of the DCCV procedures were elective (29.6%) and teaching hospitals performed significantly more DCCV than nonteaching hospitals (52.9% versus 47.1%, *p* < 0.001). Volume of DCCV procedures was distributed equally across the different regions of the United States. Incidence of in-hospital stroke was significantly lower in patients who underwent DCCV during AF related hospitalization (1% versus 2.3%, *p* < 0.0001). In-hospital mortality was also significantly lower in the AF cohort that received DCCV (0.3% versus 1.1%, *p* < 0.001). Median length of stay was 3.53 days in the DCCV group compared with 3.85 days in the non-DCCV cohort (*p* < 0.0001). Cost of hospitalization was significantly lower in patients that received DCCV as compared with those who did not (5297.89$ versus 7780.22$, *p* < 0.0001) (Table 2). On multivariate logistic regression analysis, age < 40 years, male sex, Caucasian race, private payer status, and lower CHADS2 score were independent predictors of utilization of DCCV in AF (Table 3).

## 4. Discussion

Our study has several important findings. First, using a large real world population sample, we found significant disparities in the utilization of DCCV in the United States based on race, sex, insurance status, and comorbidities. These findings are consistent with results from other studies that identify race and gender as factors contributing to disparate utilization of procedural treatments in patients with AF [4, 5]. An important finding in our study is that use of DCCV is associated with a significant lower rate of in-hospital stroke and mortality, along with decreased length of hospital stay and hospitalization costs.

Maintenance of sinus rhythm is a highly debated area in the management of AF. Data from large randomized controlled trials has shown that rhythm control does not offer any additional survival benefit over rate control in the management of AF [6, 7]. In patients with AF and congestive heart failure, the AF-CHF trial showed that restoration of sinus rhythm was not significantly more beneficial than rate control [8]. Results from these trials led to a movement promoting rate control; however, contrarians to this idea believe that factors such as poor antiarrhythmic efficacy, high risk of toxicity, and older age of the patients included in the trials override the benefits of rhythm control. With the discovery of more effective antiarrhythmic agents and procedural techniques such as AF ablation, superiority of rhythm control over rate control is postulated. An exploratory analysis in patients less than 65 years of age has shown lower all-cause mortality with rhythm control when compared with rate control [9]. Most trials comparing the two treatment strategies have followed up patients for up to 5 years showing no survival benefit with rhythm control. A Canadian study included 26130 participants with newly diagnosed AF and followed them up for up to 9 years. They discovered that mortality was higher in the rhythm control group in the first 6 months, was equal in both groups for about 4 years, and trended down in the rhythm control group as compared to rate control group after the fifth year [10]. This data predicts that, with readvent of rhythm control, DCCV and AF ablation rates are expected to trend up in the next decade [11].

Race and gender related disparity in AF therapies has been studied previously. Naderi et al. explored racial differences in AF management in hospitalized patients and found that black men were less likely to receive an ablation or DCCV procedure [5]. Bhave et al. analyzed Medicare encounter data for 517,94 patients with newly diagnosed AF and discovered that white men receive the most aggressive care [4]. In an observational study of 5333 AF patients, Dagres et al. found that, in patients with atypical or no AF related symptoms, women underwent rhythm control less frequently as compared to men [12]. While all the reasons for these disparities are not entirely clear, differences in the clinical manifestations of AF, comorbidities, and patient and provider preferences have been shown to contribute. Data from the Euro Observational Research Programme Pilot Survey on AF [12] indicates that women are oftentimes older and more symptomatic with AF than men and have a higher risk of stroke, making them more likely to receive rate control. Men, on the other hand, are more likely to develop tachycardia-induced cardiomyopathy [13], prompting more frequent use of a rhythm control strategy. Rienstra et al., in the Rate Control versus Electrical Cardioversion Study, found that women with persistent AF have greater cardiovascular morbidity and mortality when randomized to a rhythm control strategy as compared to rate control, while maintaining a similar quality of life [14]. Other factors related to treatment, access to healthcare, and affordability also play a part. African American patients have been shown to have lower rates of anticoagulation [15], prompting the use of a rate control strategy. Patients who do not have access to tertiary care centers or specialists who perform this procedure on a frequent basis miss out on the opportunity to get DCCV. Results from our study show that patients admitted to teaching hospitals are more likely to get DCCV. This could be partly explained by the availability of cardiologists and electrophysiologists with a higher level of experience. Insurance (payer) status was found to be a significant predictor for DCCV in our analysis. This is consistent with data from other studies that describe disparities in utilization and outcomes based on payer status [16, 17]. An interesting finding in our study was the relationship between comorbidities and DCCV utilization. Patients with less comorbidity such as hypertension, diabetes, congestive heart failure, and an overall lower CHADS2 score were found to be more likely to receive DCCV. Intuitively, these patients have a lower risk of stroke and greater chances of reversion as well maintenance of normal sinus rhythm, explaining our findings. Patients who underwent DCCV were found to have better outcomes in terms of fewer complications, mortality, and economic burden of hospitalization, as previously reported by Deshmukh et al. [18]. The lower rate of post-DCCV stroke in the nationwide sample is encouraging. Our results show that DCCV is an effective yet simple strategy to convert to normal sinus rhythm that can be employed in patients with AF with additional benefits of reduction in hospital mortality and hospitalization cost.

## 5. Limitations

Our study has several limitations that are inherent to large nationwide databases. NIS records discharge diagnoses and billing level data, which has potential to be miscoded leading to misrepresentation of the procedure volume. Inaccurate coding or lack of coding for comorbidities could lead to erroneous interpretations related to overall level of sickness or health. This database lacks information about clinical characteristics of the disease such as type and duration of AF, etiology, symptomatology, anticoagulant use, and imaging data, all of which are important factors considered while making treatment decisions. Lack of this data creates scope for unmatched variables between the two groups that could be contributing to the results. Due to lack of data regarding the type of AF, patients have not been substratified into groups to identify those who were not candidates for cardioversion such as paroxysmal AF or long-standing persistent atrial fibrillation. These limitations may confound study results and the improved outcomes with cardioversion may not be causal due to underlying conditions influencing its application rather than the procedure itself. Lack of hospital course and follow-up data makes it impossible to assess the degree of success or failure of the procedure in the longer term. The postprocedure complications recorded in this database are those that occur during the same admission and lack of postdischarge complications questions the long-term implications; for example, AF patients undergoing DCCV have a high risk for stroke in the first 4 weeks after the procedure and we do not have data about this beyond the in-hospital stay. A large number of patients with AF are managed in the outpatient setting, while the current study provides information pertaining to AF related hospitalization.

## 6. Conclusion

In a large nationally representative sample of patients hospitalized with AF, only a small fraction were found to undergo DCCV. Procedure trend for DCCV in AF has been fairly stable over the last 10 years while the number of hospitalizations for AF has been rising steadily. Younger patients, white patients, men, and those with fewer comorbid conditions are most likely to receive DCCV during AF related hospitalization. Patients who undergo DCCV were found to have lower rates of complications and lower length of hospital stay as well as cost of hospitalization. These findings need to be investigated in further detail at a more granular level in order to create solutions to eliminate these disparities.

## Figures and Tables

**Table 1:** Demographics, comorbidities, payer status, and hospital data for patients hospitalized for atrial fibrillation.

	No cardioversion	Cardioversion	*p* value
*Demographics*			
Age (years)			<0.0001
≤40	262580 (9.6%)	1275 (1%)	
40–64	752172 (27.6%)	42332 (34.7%)	
65–74	605721 (22.2%)	30118 (24.7%)	
≥75	1108150 (40.6%)	36644 (30.1%)	
Sex			<0.001
Men	1282442 (46.9%)	70983 (58.4%)	
Women	1453591 (53.1%)	50611 (41.6%)	
Race			<0.0001
White	2223231 (81.5%)	105241 (86.4%)	
Black	220210 (8.1%)	7225 (5.9%)	
Others	285236 (10.5%)	9374 (7.7%)	
*Comorbidities*			
Hypertension	1043744 (38.3%)	49886 (40.9%)	<0.0001
Diabetes	571796 (20.9%)	21596 (17.8%)	<0.001
Congestive heart failure	403164 (14.7%)	16330 (13.4%)	0.0012
CHADS2 score			<0.0001
0	345294	14603	
1	799072	30766	
>1	1584312	76471	
*Payer status*			<0.0001
Private	737885 (27.1%)	45040 (37%)	
Medicare	1662845 (61%)	64805 (53.2%)	
Medicaid	173315 (6.4%)	6175 (5.1%)	
Others	151140 (5.5%)	5660 (4.6%)	
*Hospital type*			<0.001
Teaching center	1064453 (39.2%)	57191 (47.1%)	
Nonteaching center	1651500 (60.8%)	64177 (52.9%)	

**Table 2:** Differences in clinical outcomes between patients hospitalized for AF based on use of cardioversion.

Outcomes	No cardioversion	Cardioversion	*p* value
Postop stroke	67897 (2.3%)	1287 (1%)	<0.0001
Cost of hospitalization ($ ± SD)	7780.22 (106.66)	5297.89 (114.21)	<0.0001
Length of stay (days ± SD)	3.85 (0.02)	3.53 (0.05)	<0.0001
In hospital mortality	30408 (1.1%)	412 (0.3%)	<0.0001

**Table 3:** Multivariate regression analysis for predictors of cardioversion in patients hospitalized for atrial fibrillation.

	Odds ratio	*p* value
Age (years)		<0.0001
<40	1.00 (Ref)	
40–64	1.07 (0.99–1.15)	
65–74	1.03 (0.94–1.12)	
≥75	0.7 (0.64–0.76)	
Sex		<0.0001
Men	1.26 (1.23–1.31)	
Women	1.00 (Ref)	
Race		<0.0001
White	1.00 (Ref)	
Black	0.66 (0.64–0.74)	
Others	0.62 (0.57–0.67)	
Hospital type		<0.0001
Teaching	1.00 (Ref)	
Nonteaching	0.58 (0.52–0.64)	
CHADS2 score		0.0027
CHADS = 0	1.00 (Ref)	
CHADS = 1	0.91 (0.86–0.97)	
CHADS > 1	0.92 (0.88–0.97)	
Diabetic	1.00 (Ref)	<0.0001
Nondiabetic	1.18 (1.14–1.23)	
Insurance		<0.0001
Private	1.00 (Ref)	
Medicare	0.84 (0.81–0.89)	
Medicaid	0.69 (0.64–0.74)	
Others	0.66 (0.59–0.74)	

## References

[1] Colilla S., Crow A., Petkun W., Singer D. E., Simon T., Liu X. (2013). Estimates of current and future incidence and prevalence of atrial fibrillation in the U.S. adult population. *American Journal of Cardiology*.

[2] Patel N. J., Deshmukh A., Pant S. (2014). Contemporary trends of hospitalization for atrial fibrillation in the united states, 2000 through 2010: implications for healthcare planning. *Circulation*.

[3] January C. T., Wann L. S., Alpert J. S. (2014). 2014 AHA/ACC/HRS Guideline for the management of patients with atrial fibrillation: executive summary: a report of the American College of Cardiology/American Heart Association Task Force on practice guidelines and the Heart Rhythm Society. *Journal of the American College of Cardiology*.

[4] Bhave P. D., Lu X., Girotra S., Kamel H., Vaughan Sarrazin M. S. (2015). Race and gender related differences in care for patients newly diagnosed with atrial fibrillation. *Heart Rhythm*.

[5] Naderi S., Rodriguez F., Wang Y., Foody J. M. (2014). Racial disparities in hospitalizations, procedural treatments and mortality of patients hospitalized with atrial fibrillation. *Ethnicity and Disease*.

[6] Wyse D. G., Waldo A. L., DiMarco J. P. (2002). A comparison of rate control and rhythm control in patients with atrial fibrillation. *The New England Journal of Medicine*.

[7] Hagens V. E., Vermeulen K. M., TenVergert E. M. (2004). Rate control is more cost-effective than rhythm control for patients with persistent atrial fibrillation—results from the RAte Control versus Electrical cardioversion (RACE) study. *European Heart Journal*.

[8] Talajic M., Khairy P., Levesque S. (2010). Maintenance of sinus rhythm and survival in patients with heart failure and atrial fibrillation. *Journal of the American College of Cardiology*.

[9] Rolf S., Kornej J., Dagres N., Hindricks G. (2015). What can rhythm control therapy contribute to prognosis in atrial fibrillation?. *Heart*.

[10] Ionescu-Ittu R., Abrahamowicz M., Jackevicius C. A. (2012). Comparative effectiveness of rhythm control vs rate control drug treatment effect on mortality in patients with atrial fibrillation. *Archives of Internal Medicine*.

[11] Pothineni N. V. K., Deshmukh A., Pant S. (2014). Complication rates of atrial fibrillation ablations: comparison of safety outcomes from real world to contemporary randomized control trials. *International Journal of Cardiology*.

[12] Dagres N., Nieuwlaat R., Vardas P. E. (2007). Gender-related differences in presentation, treatment, and outcome of patients with atrial fibrillation in Europe: a report from the Euro heart survey on atrial fibrillation. *Journal of the American College of Cardiology*.

[13] Potpara T. S., Marinkovic J. M., Polovina M. M. (2012). Gender-related differences in presentation, treatment and long-term outcome in patients with first-diagnosed atrial fibrillation and structurally normal heart: the Belgrade atrial fibrillation study. *International Journal of Cardiology*.

[14] Rienstra M., Van Veldhuisen D. J., Hagens V. E. (2005). Gender-related differences in rhythm control treatment in persistent atrial fibrillation: data of the rate control versus electrical cardioversion (RACE) study. *Journal of the American College of Cardiology*.

[15] Thomas K. L., Piccini J. P., Liang L. (2013). Racial differences in the prevalence and outcomes of atrial fibrillation among patients hospitalized with heart failure. *Journal of the American Heart Association*.

[16] Kapoor J. R., Kapoor R., Hellkamp A. S., Hernandez A. F., Heidenreich P. A., Fonarow G. C. (2011). Payment source, quality of care, and outcomes in patients hospitalized with heart failure. *Journal of the American College of Cardiology*.

[17] Brinjikji W., El-Sayed A. M., Kallmes D. F., Lanzino G., Cloft H. J. (2015). Racial and insurance based disparities in the treatment of carotid artery stenosis: a study of the Nationwide Inpatient Sample. *Journal of NeuroInterventional Surgery*.

[18] Deshmukh A., Pant S., Kumar G., Bursac Z., Paydak H., Mehta J. L. (2012). Comparison of outcomes of weekend versus weekday admissions for atrial fibrillation. *American Journal of Cardiology*.

